# Formaldehyde

**Published:** 1898-09

**Authors:** 


### Formaldehyde.

The *American Microscopical Journal* says: "The credit of the discovery of the powerful antiseptic properties of formaldehyde and its practical application is due to A. Frillat, who in 1888 first noticed its preserving action on samples of wine, and in 1891 made public his experiments, showing it to possess antiseptic properties much superior to all non-toxic organic antiseptics then known."

